# Molecular causes of an evolutionary shift along the parasitism–mutualism continuum in a bacterial symbiont

**DOI:** 10.1073/pnas.2005536117

**Published:** 2020-08-19

**Authors:** Paul Herrera, Lisa Schuster, Cecilia Wentrup, Lena König, Thomas Kempinger, Hyunsoo Na, Jasmin Schwarz, Stephan Köstlbacher, Florian Wascher, Markus Zojer, Thomas Rattei, Matthias Horn

**Affiliations:** ^a^Centre for Microbiology and Environmental Systems Science, University of Vienna, 1170 Vienna, Austria

**Keywords:** transmission mode, host–microbe interaction, virulence evolution, intracellular bacteria, chlamydia

## Abstract

Symbiosis with microbes is a ubiquitous phenomenon with a massive impact on all living organisms, shaping the world around us today. Theoretical and experimental studies show that vertical transmission of symbionts leads to the evolution of mutualistic traits, whereas horizontal transmission facilitates the emergence of parasitic features. However, these studies focused on phenotypic data, and we know little about underlying molecular changes at the genomic level. Here, we combined an experimental evolution approach with infection assays, genome resequencing, and global gene expression analysis to study the effect of transmission mode on an obligate intracellular bacterial symbiont. We show that a dramatic shift in the frequency of genetic variants, coupled with major changes in gene expression, allow the symbiont to alter its position in the parasitism–mutualism continuum depending on the mode of between-host transmission. We found that increased parasitism in horizontally transmitted chlamydiae residing in amoebae was a result of processes occurring at the infectious stage of the symbiont’s developmental cycle. Specifically, genes involved in energy production required for extracellular survival and the type III secretion system—the symbiont’s primary virulence mechanism—were significantly up-regulated. Our results identify the genomic and transcriptional dynamics sufficient to favor parasitic or mutualistic strategies.

## Significance

Symbiotic relationships with microbes are ubiquitous among living beings and can be parasitic, such as in bacterial pathogens, or mutualistic, as in beneficial microbiomes. Among other factors, the outcome of microbe–host relationships is determined by the mode of symbiont transmission from host to host. Here we describe how bacterial symbionts increased in infectivity and virulence toward their amoeba host when transmission to a new host was essential for survival. The enhanced parasitism is a result of genomic changes and a pronounced switch of gene expression altering the symbionts’ mechanisms for host interaction. Our study provides both a molecular explanation as well as a blueprint for how changes in gene expression are sufficient to confer enhanced parasitism in microbes.

The relationship between the partners in microbial symbioses have been described as existing along a parasitism–mutualism continuum (1), and the dynamics of this continuum are dependent upon numerous genotypic and environmental factors (2–6). The transmission mode of viruses, bacteria, and eukaryotic parasites is a key factor shaping the ecology and evolution of symbiotic associations (7–10). In the case of vertical transmission (VT), symbionts are inherited from the host parent to offspring. By contrast, in horizontal transmission (HT), symbiont transmission is not linked to host reproduction, but instead symbionts infect new host lineages. The mode of symbiont transmission among host generations has important evolutionary ramifications: while VT aligns the evolutionary interest of host and symbionts, thus selecting for benign or even mutualistic symbionts, HT does not penalize parasitic strategies, because symbionts are associated with a new host individual every round (11–14). As a consequence, HT promotes the evolution of virulence (15–17), broadly defined as the decrease in host fitness as a result of infection, which can ultimately lead to host mortality (18–21). Consistent with this notion, experimental manipulation in the laboratory has demonstrated that propagating symbionts via either HT or VT will select for parasitic or mutualistic traits, respectively (19, 22–24). However, the molecular adaptations causing the observed shifts in the parasitism–mutualism continuum remain elusive.

Here, we take advantage of the bacterial symbiont *Parachlamydia acanthamoebae* and its protist host *Acanthamoeba* sp. as a tractable model system. Chlamydiae, which represent the most ancient known group of obligate intracellular bacteria, all share a characteristic biphasic developmental cycle alternating between two distinct morphological and physiological stages: the elementary body (EB) survives (but cannot replicate) outside eukaryotic host cells and infects new hosts, whereas the reticulate body (RB) replicates inside the host cell within a host-derived vacuole (25–27). Chlamydiae are among the most successful bacterial pathogens of humans. Their environmental representatives (28), such as the one used here, are ubiquitous in the environment and live primarily within unicellular protists like amoeba. During host cell division this symbiont can be vertically transmitted from parent to daughter cells (VT), but can also readily infect naive hosts (HT). This mixed transmission mode (29) represents an ideal starting point for experimentally manipulating the transmission of symbionts among host generations (i.e., VT or HT), and in this way identify the molecular changes that arise in response to the different selection regimes.

## Results and Discussion

### Design of Evolution Experiment.

**Fig. 1.**
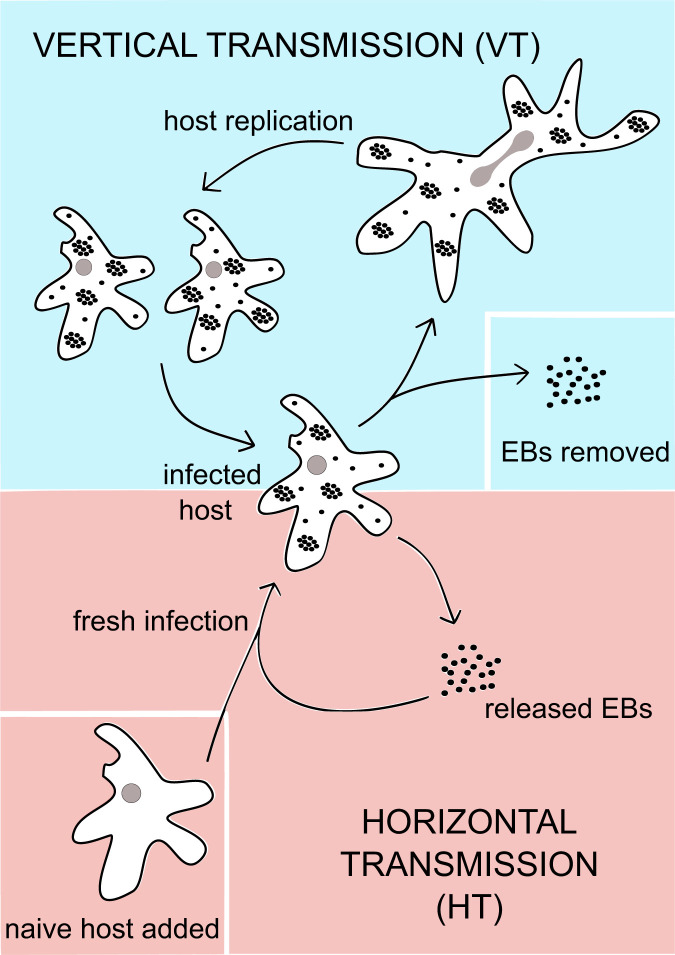
The *Parachlamydia/Acanthamoeba* model and the design of the evolution experiment. Two separate selection regimes were established to select for HT and VT, both originating from the same initial ancestral amoeba–symbiont population. In VT (*Top*), amoebae that were fully infected with the symbiont were maintained in liquid culture. Daily removal of the liquid medium was carried out to remove released EBs, minimizing the potential for HT. No new amoebae were added to the cultures, and the bacterial symbionts were passed on from parent cell to daughter cell. This was performed for 14 mo, equivalent to 525 symbiont generations. In HT (*Bottom*), EBs were allowed to accumulate over a week in the liquid medium, after which they were isolated and used to infect naive host cells. Infected amoebae were discarded at the end of the week. Fresh infections, by way of HT, were required for the symbionts to be maintained in the population. This was performed for 14 mo, equivalent to 560 symbiont generations.

In our VT regime, fully infected amoebae were allowed to replicate continuously and no naive hosts were added throughout the experiment (Fig. 1). EBs, the infectious chlamydial stage essential for horizontal transmission, were removed daily. This ensured that the extent of HT was reduced to a minimum and that the chlamydiae were predominantly transferred vertically from parent to daughter cells. In the HT regime EBs, which had been released from their amoeba host cells, were collected weekly and used to freshly infect naive amoebae. VT was minimized and HT was essential in this regime as all infected amoeba cells were removed at the end of the week, with only the infectious EBs being isolated and used for fresh infections. Both regimes were initiated from the same ancestral *Chlamydia* and amoeba populations and maintained for a duration of 14 mo, which corresponded to about 500 to 600 symbiont generations (*SI Appendix*, *SI Text*).

### Fitness Assays Reveal a Shift toward Parasitism under HT Conditions.

**Fig. 2.**
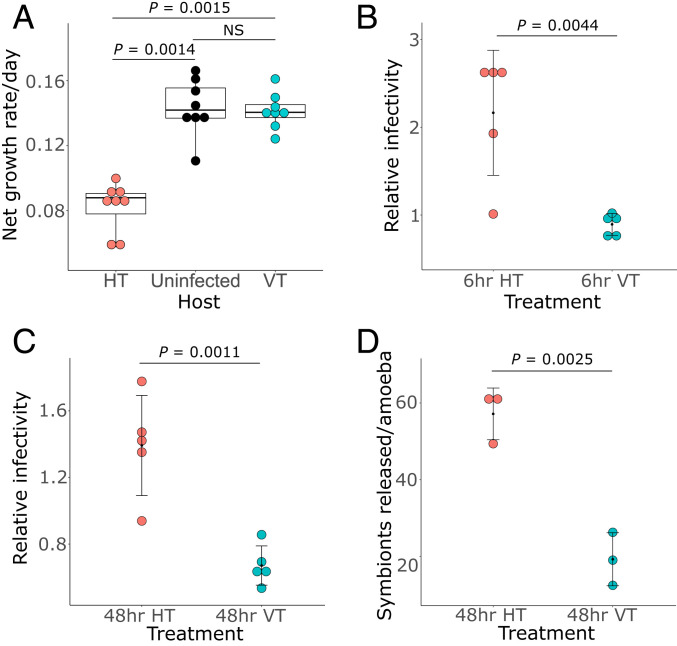
Symbionts isolated from the HT regime are more virulent than symbionts from the VT regime. (*A*) Amoeba populations (*n* = 8) that were either fully infected with symbionts from the HT (red dots) or VT (blue dots) treatment, or uninfected (black dots), were seeded at a concentration of 2 × 10^4^ cells per milliliter and incubated at 20 °C. The growth of the amoebae after 7 d was quantified and used to calculate the net growth rate per day. In the box plots, the interquartile range (IQR) between the first and the third quartiles is indicated by the box, while vertical lines extend to a distance of 1.5 × IQR from the first or third quartile. The horizontal line within the box represents the median. The rates were compared using a Kruskal–Wallis test (χ^2^ [2] = 15.425, *P* < 0.0005) followed by Dunn’s multiple comparison post hoc test. NS, not significant. All growth rates are provided in Dataset S1. (*B*–*D*) Naive amoebae at a concentration of 10^5^ cells per milliliter were infected with symbionts isolated from the HT (red dots) or VT (blue dots) treatment with an MOI of 5. The relative infectivity was determined by dividing the total symbiont number per milliliter (inside and outside host cells) of the particular treatment (*n* = 5) by that of the ancestral symbiont after 6 hpi (*B*) and 48 hpi (*C*). Data represent mean ± SD of the mean. Numbers of bacteria were compared using an unpaired two-tailed Student’s *t* test for *B*, *t*(8) = 3.930, *P* < 0.005 and *C*, *t*(8) = 4.989, *P* < 0.005). All relative infectivity values are provided in Dataset S1. (*D*) Total numbers of symbionts released outside the host cells per amoeba for each treatment (*n* = 3) were compared using an unpaired two-tailed Student’s *t* test, *t*(4) = 6.792, *P* < 0.005. Data represent mean ± SD of the mean. All individual values for total numbers of symbionts released are provided in Dataset S1.

To understand how symbiont and host fitness changed throughout the experiment, infection experiments were carried out to quantify growth parameters of both host and symbiont. Naive amoebae that were infected with symbionts isolated at the end of the experiment from the VT treatment displayed a net growth rate that was statistically indistinguishable from the one of uninfected amoebae (Fig. 2*A*). This observation indicated that vertically transmitted symbionts had minimal negative effects on the host. On the other hand, the net growth rate of naive amoebae freshly infected with symbionts isolated at the end of the experiment from the HT treatment was roughly half the net growth rate of both uninfected amoebae and amoebae from the VT treatment (Fig. 2*A*). The observed pronounced decrease in host fitness when challenged with HT versus VT symbionts suggests a strong shift toward parasitism in horizontally transmitted symbionts.

The fitness of chlamydial symbionts during these infection experiments was quantified at two stages during the infection process. At 6 h postinfection (hpi) EBs have successfully entered the host cell, initiated differentiation to RBs, and started the establishment of their intracellular niche. This time point thus determines the ability of the symbionts to infect the amoeba host at the start of the experiment. At 48 hpi, one chlamydial developmental cycle is complete, and the ability of the symbionts to rapidly multiply and subsequently be released back into the medium can be determined. Symbionts originating from the HT treatment were highly infectious from the start of the fitness assay: already 6 hpi, the symbiont numbers in amoebae infected with HT chlamydiae were double that of the VT treatment (Fig. 2*B*). These elevated symbiont numbers were maintained until the completion of one developmental cycle (i.e., 48 hpi) (Fig. 2*C*). Moreover, a significantly higher number of HT symbionts compared to VT symbionts was released per host cell at this time point (Fig. 2*D*). These results demonstrate that chlamydiae isolated from the HT regime infected their host more efficiently than the ones isolated from the VT regime. This includes the initial uptake, formation, and replication of RBs and release of infectious EBs into the supernatant. The increased symbiont fitness reflects the observed reduction in host fitness (Fig. 2*A*), together showing a pronounced shift toward parasitism under HT conditions. An increase in symbiont virulence and parasitism through HT meets theoretical expectations (15–17). Although these predictions have been previously demonstrated for viruses and eukaryotic parasites, only very few studies exist so far that verified the link between transmission mode and the level of virulence in bacteria (30, 31).

### Ancestral Variants Increased in Frequency in the HT Symbionts.

**Fig. 3.**
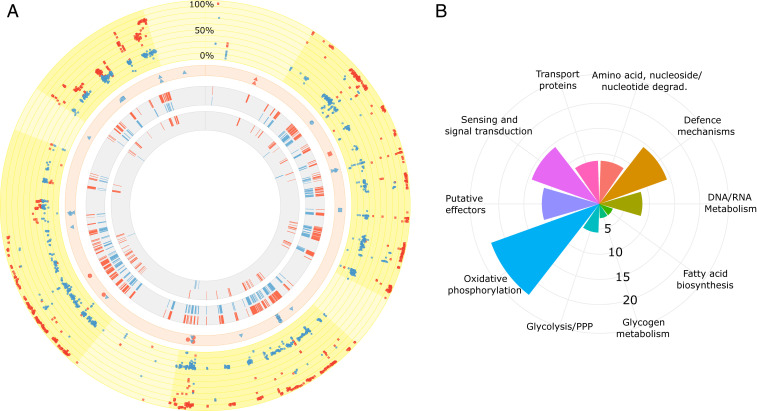
A majority of genetic variants strongly increased in frequency in the symbionts from the HT regime and were associated with differentially expressed genes. The circular plots represent the symbiont genome. (*A*) In all four rings, red indicates HT and blue indicates VT. The outermost ring depicts a scatterplot for the entire symbiont genome with all of the 1,436 ancestral genetic variants identified in the HT and VT symbiont populations. A large majority of the HT variants reach very high frequencies above 80%. One symbol represents a single variant. Lighter yellow sections represent conserved regions of the genome that possess a high proportion of housekeeping genes and are less affected by variants (i.e., genes involved in replication, translation, central carbon metabolism, energy production and conversion, metabolism, and transport). The second ring shows the location of the 33 novel variants (9 = HT; 24 = VT) identified throughout the genome (circle, intergenic; triangle, genic; and rectangle, potential promoter region). The third ring depicts those genes from either treatment (354 = HT; 248 = VT) that 1) harbored variants with a pronounced difference in frequency (±15%) between the two treatments, and 2) were differentially expressed in the respective treatment. The innermost ring depicts those genes (112 = HT; 8 = VT) from the third ring that were solely differentially expressed in one but not the other treatment. Genome visualization was carried out with Circos (32). (*B*) Coxcomb chart summarizes the known functional categories of the 112 unique genes differentially expressed in the HT symbiont population. Axis indicates the percentage of genes affected per functional category. All individual values are provided in Dataset S3.

Our phenotypic data suggest that the increase in symbiont virulence is a consequence of changes in the bacterial developmental cycle, emphasizing the efficiency of uptake and the generation and release of infectious bacteria into the environment. As a next step, we set out to investigate the molecular basis of these adaptations. The symbiont genome consists of a single circular chromosome (3,072,383 bp) with 2,618 genes (27). Pool sequencing of bacteria isolated from either treatment at the end of the experiment after 14 mo allowed us to identify genomic changes that occurred in the symbiont populations during the experiment (Fig. 3*A*). We identified 9 novel variants in the horizontal treatment and 24 novel variants in the vertical treatment, with many of them at low frequencies and none affecting genes involved in critical cellular functions (Dataset S2). Conversely, standing genetic variation in the initial ancestral population—the founder *Chlamydia* and amoeba populations from which both regimes were initiated—appeared to be particularly important for the observed evolutionary changes. From a total of 1,436 ancestral variants, mainly single nucleotide polymorphisms (SNPs), spread around the genome, 1,161 variants (80.8% of all variants; Dataset S2) pronouncedly increased in frequency in the HT symbiont population as compared to the VT population, with a large majority of these variants reaching very high frequencies above 80% (Fig. 3*A*). On the other hand, only 134 of these variants (9.3% of all variants; Dataset S2) increased in frequency in the VT population relative to the HT population. The strong shift in variant frequencies under HT conditions revealed a major change in the symbiont population concomitant with the observed phenotypic change in parasitism during our experiment.

In order to investigate this shift in variant frequencies, we focused on the set of genes that appeared most different between the two experimental conditions. For this, we focused on genes that included variants with a greater than 15% differential in frequency. These variants were distributed in a total of 446 genes accounting for 17% of the protein coding sequences, without apparent mutational hot spots. To better understand how these genes are related to the observed phenotypes, we tested whether they are involved in specific cellular functions. Apart from considering the identity of the affected genes, the variants were divided into numerous subgroups, based on whether they increased in frequency in the HT or the VT regime, as well as what type of variant it was (synonymous, nonsynonymous, or promoter region). Surprisingly, no statistically significant enrichments with respect to their predicted cellular functions were identified in any of these groups. Therefore, although genome resequencing of the symbionts revealed a major population shift and identified hundreds of genetic variants that increased in frequency under HT, the genome sequencing data alone does not provide any clues about the molecular basis underlying the observed phenotypes. We thus next asked whether insights into gene expression dynamics could help understand the difference in parasitism between HT and VT symbionts. Using bacteria isolated at the end of the experiment from either selection regime, we performed Illumina-based RNA sequencing (RNA-Seq) analysis on symbionts infecting naive amoebae isolated at three time points that mark crucial developmental events during infection: 2 hpi, representing the start of infection and EB-to-RB transition; 24 hpi, as the peak of replication and RB activity; and released EBs accumulated at 7 d postinfection, representing the new generation of symbionts ready to infect new host cells.

### A Large Contrast in Temporal Gene Regulation.

**Fig. 4.**
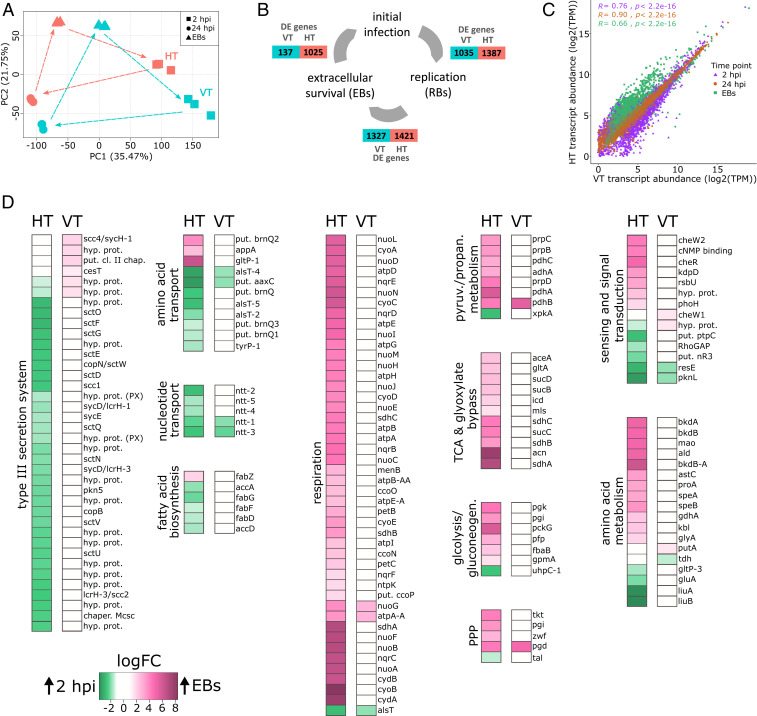
Genes involved in extracellular survival and virulence were up-regulated in HT symbionts. (*A*) Principal component (PC) analysis plot for RNA-Seq gene expression data at three time points of symbionts from the HT or VT treatment that were collected from amoeba host cells (2 hpi and 24 hpi) or from the medium (released EBs) (*n* = 3). (*B*) An overview of the total number of differentially expressed (DE) genes for either treatment between two consecutive time points that marked crucial developmental events during infection (*n* = 3). Genes were considered DE if their expression changed twofold with a FDR smaller than or equal to 0.05. (*C*) Scatterplot showing the correlation between transcript abundance in the two treatments at each time point (*n* = 3). Each dot on the scatterplot represents a single gene. Kendall’s tau coefficient at each time point is shown on the plot. (*D*) Heat maps depict those metabolic pathways and T3SS loci with marked differences between the HT and VT treatments with respect to logFC of genes that were differentially expressed between released EBs and the 2 hpi (FDR of 0.05) (*n* = 3). A logFC below 1 (no differential expression) is shown in white, significant up-regulation in released EBs is shown in pink, and significant up-regulation at 2 hpi is shown in green. The stronger the color, the stronger the gene expression change between the two time points. All logFC values are provided in Dataset S8.

Global gene expression analysis showed a highly dynamic transcriptional landscape during infection, including pronounced temporal signatures consistent with previous findings for chlamydial symbionts (33). Strikingly, the gene expression profiles of VT and HT symbionts were very different at all time points analyzed, indicating marked changes in temporal gene regulation (Fig. 4*A* and Dataset S4). To dissect these temporal differences, we next focused more specifically on which genes were differentially expressed between two consecutive time points (Dataset S5). We observed the major discrepancy between HT and VT symbionts when comparing the number of differentially expressed genes between released EBs and 2 hpi, reflecting early events during infection (Fig. 4*B*). In the symbionts from the VT regime, only 137 genes were differentially expressed between these two early time points, whereas 1,025 genes were differentially expressed in the symbionts from the HT regime (Fig. 4*B*). We next investigated whether the identified differentially expressed genes are associated with the set of genes that were characterized by a major shift in variant frequency in our previous analysis. In fact, from this set of 446 genes, 354 and 248 were differentially expressed at some point during the developmental cycle of HT and VT symbionts, respectively (Fig. 3*A* and Dataset S6). When we narrowed down our search in these two groups to those genes that were solely differentially expressed in one regime, we identified only 8 unique genes for the VT symbionts (Dataset S7). By contrast, a total of 112 unique genes were identified for the HT symbionts (Fig. 3*A* and Dataset S7). Given that the latter genes exhibited significant changes in variant frequencies and were solely differentially expressed in HT symbionts, they are likely associated with the increase in virulence in the symbiont population from the HT regime. This notion is further supported by the fact that the function of these genes has been previously implicated in chlamydial survival and virulence (33, 34). This included oxidative phosphorylation, sensing and signal transduction, transport, as well as the type III secretion system (T3SS) (Fig. 3*B*).

### Pronounced Differences Observed at the Single Gene and Pathway Levels.

How were these and other pathways and functional categories represented in the distinct temporal expression patterns of HT and VT symbionts? To address this, we analyzed gene expression levels and gene regulation of selected pathways in detail. We consistently observed that gene expression levels were broadly similar for both HT and VT symbionts during replication (i.e., at 24 hpi; Fig. 4*C* and Dataset S4). The most highly expressed genes at this time point are involved in cellular processes such as cell division and peptidoglycan synthesis, DNA replication and translation, and metabolic activity (lipid and amino acid transport and metabolism), together representing hallmarks of the chlamydial replicative stage (26, 35) (*SI Appendix*, Figs. S1–S3 and Dataset S9). In contrast, pronounced differences between HT and VT chlamydiae at the single gene and pathway levels were apparent at the onset of infection (2 hpi) and even more so at the EB stage (Fig. 4*D*). This included a significant up-regulation of structural components, effectors, and chaperones of the T3SS early in infection in HT symbionts, while this system was not differentially expressed at this time point in VT symbionts (Fig. 4*D*). The T3SS was already present in the last common ancestor of all extant chlamydiae and is a primary and evolutionarily well-conserved chlamydial virulence factor (36, 37). Another set of genes up-regulated early in HT but not VT functions in fatty acid biosynthesis (Fig. 4*D*). As obligate intracellular bacteria, chlamydiae rely on subversion of diverse host cell metabolites, yet de novo fatty acid and lipid biosynthesis is considered essential for replication (38). The largest difference in gene expression between HT and VT symbionts was observed in the released EBs, the extracellular stage required for survival and infection of new host cells. HT symbionts showed pronounced up-regulation of genes involved in oxidative phosphorylation, glycolysis/gluconeogenesis, the citric acid cycle, pyruvate/propanoate metabolism, and the pentose phosphate pathway—all involved in the utilization of carbon substrates and energy conservation. This pattern was not detectable in EBs from the VT regime (Fig. 4*D*). However, it resembles the gene expression profile of EBs in closely related chlamydial symbionts for which glucose metabolism and respiration has been shown to be important at the infectious stage (33, 34).

## Conclusions

Our gene expression analysis supports a scenario in which HT symbionts that invade the host as EBs were better adapted to both survival in the extracellular environment and infection of their amoeba host than VT symbionts. The more virulent HT phenotype as observed in the symbiont fitness assays (Fig. 2) can be explained by a more pronounced expression of genes and pathways known to be important for EB maturation and extracellular persistence. Outside the host cell, access to nutrients, such as glucose and respiration, is crucial to maintain infectivity (34, 39, 40), and it is exactly the genes involved in these processes that are affected by both strong variant shifts and pronounced differential expression (Figs. 3 and 4). When encountering a new host cell, secretion of effector proteins is a crucial initial step of infection and required for host cell manipulation to establish the intracellular niche (41). The main mechanism to achieve this, the chlamydial T3SS, is more strongly up-regulated in HT symbionts at 2 hpi, providing a likely reason for the significantly higher infectivity in these compared to VT symbionts (Fig. 2*B*).

VT symbionts greatly resembled the founder ancestral population, with respect to both phenotype and genotype. When shown relative to each other at 6 hpi and at 48 hpi, the infectivity of the VT symbionts did not differ greatly from the infectivity of the ancestral population (Fig. 2 *B* and *C*). Furthermore, the vast majority of ancestral variant frequencies did not change much in the VT population, with novel mutations largely remaining at low frequencies (Fig. 3*A* and Dataset S2). Conversely to the HT symbionts, those genes and pathways known to be essential for extracellular survival and infection were not strongly up-regulated in the VT symbionts, and this likely determined their lower infectivity. The attenuated character of the VT population makes the symbionts better suited for long-term coexistence with their amoeba hosts, and this regime more closely resembles the transmission mode of these bacteria under standard laboratory conditions, where the symbionts do not encounter naive hosts.

In nature one expects a mixed transmission mode for these environmental chlamydiae, where both HT and VT co-occur simultaneously. Numerous factors interact together, some of which favor one transmission mode over the other. One such factor likely playing an important role in the environment is host cell density. Where host individuals are common and encountering new hosts is not limiting, HT would increase because selection on host fitness is relaxed. Furthermore, an increase in HT results in higher symbiont diversity within hosts leading to within-host competition which, in turn, promotes more virulence (20, 42). However, at low host cell density, VT is more likely to dominate because host fitness is particularly crucial for symbiont survival, selecting for attenuated phenotypes. We argue that in our system, virulence (i.e., host fitness costs due to the infection) is ultimately a compromise between within-host replication and host mortality. In fact, the tradeoff hypothesis, discussed and challenged for the past three decades, proposes that transmission will have a strong effect on virulence evolution (43, 44). In the model system investigated here, the symbionts are required to strike an optimal balance between being more benign (VT population) and more parasitic (HT population), and this will vary depending on the complex web of interactions in the surrounding environment.

In conclusion, we have shown that conditions favoring HT led to increased parasitism and higher infectivity in a bacterial symbiont related to major pathogens of humans. While predicted by theory (11, 15–17), experimental evidence for bacteria is sparse (30, 31, 45). Here, the observed shift toward parasitism occurred within a short time frame and was mainly driven by a change in standing genetic variation, i.e., selection for several hundred SNPs that occurred in the original symbiont population. The parasitic phenotype was characterized by an extensive adjustment of gene expression, mainly affecting functions important at the infectious stage of the symbiont and required for host cell manipulation. The experimental and molecular evidence presented in this study contributes to a better understanding of how conditions affecting transmission mode can lead to the emergence of bacterial parasitism.

## Materials and Methods

### Evolution Experiments.

The same ancestral population of *Acanthamoeba* sp. UWC1 containing *P. acanthamoebae* UV-7 was used in the two different regimes. This population had been maintained at 20 °C in TSY medium (30 g/L trypticase soy broth, oxoid, 10 g/L yeast extract; pH 7.3). Cultures were regularly screened by fluorescence in situ hybridization and 4′,6′-diamidino-2-phenylindole (DAPI) staining (0.1 µg/mL) to exclude contamination.

In the VT regime, amoebae that were fully infected with the symbiont were maintained in 25 cm^2^ cell culture flasks (Nalge Nunc International) with 10 mL TSY medium at 20 °C. The medium was replaced daily, ensuring that as much liquid as possible, containing released EBs and cell debris, was removed. New amoebae were never added to the cultures, and the bacterial symbionts were passed on from parent cell to daughter cell. This was performed for 14 mo, equivalent to 525 symbiont generations. Amoeba–*Chlamydia* samples from the end of the VT experiment were stored with dimethyl sulfoxide as a cryoprotectant in liquid nitrogen. These samples were later used to isolate symbionts for the fitness assays.

At the start of every week in the HT regime, 3 mL TSY medium, 2 mL of a suspension of naive amoeba cells (the appropriate number of amoebae was predetermined during the initial infection cycles), and 2 mL of filtered supernatant (containing released EBs) from the previous week’s population were mixed together in 25 cm^2^ cell culture flasks. The mixture was allowed to stand for 30 min at room temperature, followed by centrifugation (1,000 rpm, 15 min, 23 °C) so as to synchronize the infection. The medium was then exchanged by pipetting, after which the cultures were allowed to grow for a week at 20 °C. Amoebae were completely infected by the end of the week, with many EBs being released in the medium. Infected amoebae were then discarded and the filtered supernatant was used to infect naive host cells.This was performed for 14 mo, equivalent to 560 symbiont generations. EBs from the end of the HT experiment were stored in SPG buffer (75 g/L sucrose, 0.52 g/L KH_2_PO_4_, 1.53 g/L NaHPO_4_·7H_2_O, 1.53 g/L Na_2_HPO_4_·2H_2_O, 0.75 g/L glutamic acid; pH 7.2) at −80 °C. These EBs were later used in the fitness assays.

### Fitness Assays.

To measure the effect that the HT and VT symbionts had on growth rate of host cells, amoeba populations that were either fully infected with symbionts isolated from the HT or VT treatment, or uninfected, were seeded in 1 mL TSY medium in 24-well plates (Thermo Fisher Scientific) at a concentration of 2 × 10^4^ cells per milliliter and incubated at the HT/VT regime temperature of 20 °C. The growth of the amoebae after 7 d was quantified by counting the number of host cells in a Neubauer chamber (VWR International). The initial and final cell numbers were used to calculate the net growth rate per day: ln(N_t_ − N_0_)/t; N_t_ is the final cell concentration, N_0_ is the initial cell concentration, and t is 7 d (46).

To quantify the virulence of the bacteria, naive amoebae were infected with symbionts purified from the HT or VT treatment at the end of the experiment, which was conducted as follows. Culture supernatant was filtered through 1.2-µm syringe filters (Sartorius) to remove residual host cells. Bacteria were collected by centrifugation (10,000 rpm, 10 min, 4 °C), resuspended in precooled SPG buffer, homogenized using a 21-gauge injection needle (B. Braun), and stored at −80 °C in SPG buffer. For quantification of purified EBs, 10-µL cell suspensions in 10 mL Page’s amoeba saline (PAS) (0.12 g/L NaCl, 0.004 g/L MgSO_4_·7H_2_O, 0.004 g/L CaCl_2_·2H_2_O, 0.142 g/L Na_2_HPO4, 0.136 g/L KH_2_PO4) were filtered onto a polycarbonate membrane with a pore size of 0.2 µm (EMD Millipore); cells were stained with 0.1 µg/mL DAPI and counted using an epifluorescence microscope (Axioplan 2 imaging; Carl Zeiss).

Symbiont-free amoebae were harvested right before infection and an aliquot was counted in a Neubauer chamber. The amoeba cell suspension in TSY was seeded in the required amount of wells (1 mL/well) in a 24-well plate at a concentration of 10^5^ amoebae/mL. The plate was then incubated at 20 °C for 1 h to allow the host cells to attach prior to infection. EBs for the samples to be tested were thawed at 37 °C in a water bath and added in triplicate to wells in the 24-well plate at a multiplicity of infection (MOI) of 5, as this was predetermined to be the best MOI to observe differences in infectivity between samples. The well plates were then incubated for 15 min at room temperature, followed by centrifugation (1,000 rpm, 15 min, 23 °C) so as to synchronize the infection. The medium was then changed so as to remove any residual EBs. This was done by removing 1 mL TSY per well by pipetting, and then readding 1 mL of fresh TSY. Wells were then sampled at 6 hpi and 48 hpi, with the latter time point being divided into supernatant fraction and amoeba fraction. Quantification of symbionts was then carried out by qPCR (see below). The relative infectivity was determined by dividing the total symbiont number per milliliter of the particular treatment (HT/VT) by that of the ancestral symbiont.

### qPCR.

We produced a standard for the absolute quantification of members of the *Chlamydiales* by qPCR assay with the primer pair panCh16F2_mod (5′-CCGCCAACAYTGGGACT-3′)/panCh16R2_mod (5′-GKAGGTRGCCGCYGCTTCTTTAC-3′) (both modified from ref. 47) targeting the *Chlamydiales* 16S rRNA gene. This qPCR standard consisted of a linear DNA molecule containing the target sequence of the primer pair that was used in the qPCR assays flanked by 90- and 76-bp regions on each end to allow the qPCR polymerase to attach. Briefly, the standard was produced by amplifying the target sequence by PCR, cloning into the pCR4TOPO vector using the TOPO-TA Cloning Kit for Sequencing by Thermo Fisher Scientific, transforming the resulting plasmids into One Shot TOP10 chemically competent *Escherichia coli* cells, and finally purifying and pooling using the QIAquick PCR Purification Kit from Qiagen.

DNA from the amoeba–*Chlamydia* cultures was isolated using the DNeasy Blood & Tissue Kit (Qiagen). All qPCR assays were performed in 16 µL, with iQ SYBR Green Supermix (Bio-Rad Laboratories), 0.25 μM concentrations of each primer (both modified from ref. 47), molecular biology grade water (Merck KGaA), and 2 μL of DNA sample using a CFX96 Touch Real-Time PCR Detection System from Bio-Rad. The cycling conditions were 3 min at 95 °C, followed by 40 cycles of 15 s at 95 °C, 30 s at 60 °C, and 30 s at 72 °C. The results of the qPCR runs were analyzed using the CFX Manager 3.1 from Bio-Rad. The melt curves were checked for any abnormalities. The absolute number of 16S-rRNA genes in the samples was determined by running a serial dilution of the standard sequences of known concentration in every qPCR run and comparing the amplification of the samples to the linear regression (*R*^2^ > 0.99 in all cases) produced by the standards. All samples were run in triplicate and molecular biology grade water was used as a negative PCR control.

### DNA Extraction and Sequencing.

Frozen amoeba–*Chlamydia* cultures from the founder population as well as from the end of the experiment of the VT regime were thawed in a water bath at 35 °C for 2 min and then pipetted into 25-cm^2^ culture flasks with 10 mL TSY to allow sufficient growth. After 1 wk, these cultures were then upscaled into 175-cm^2^ flasks (Nalge Nunc International) for another week so as to increase the quantity of EBs that could be isolated. Frozen EBs from the HT regime at the end of the experiment were used to infect naive amoeba cells and upscaled in the same way as the VT regime. EB purification from the supernatant was done as described previously for both regimes. Isolation of genomic DNA was then carried out on these isolated EBs using a cetyl trimethylammonium bromide-based extraction method (48). Library preparation and pool sequencing of symbiont populations were performed at the Vienna BioCenter Core Facilities (VBCF) next-generation sequencing (NGS) unit (https://www.viennabiocenter.org/facilities/). Illumina Genome Analyzer and HiSeq2000 instruments were used to generate paired-end reads of ∼125 bases according to standard procedures, achieving the desirable coverage of 300- to 600-fold.

### Sequence Read Processing and Mapping.

The quality filtering and trimming of the sequenced reads was done by Prinseq-lite (v0.19.5) (49) and Trimmomatic (v0.32) (50) and applied as follows. First, a sliding window with size 10 removed any bases with lower quality than 20 starting from the 3′ end by removing the read part containing the low-quality bases. Any reads shorter than 40 nucleotides were also removed. We discarded any low-quality read that had an average Phred score below 30. Only read pairs were kept. These reads were mapped against the reference genome sequence of *P. acanthamoebae* (27) (NCBI RefSeq accession no. NC_015702.1) using the Burrows–Wheeler aligner (BWA) (v0.7.5a) (51) with standard settings and stored as bam files. For conversions from sam to bam files and from bam to fastq files (as Cortex_var input), we used SAMtools (v0.1.18) (52) and Picard Tools (v1.92, broadinstitute.github.io/picard/).

### Variant Prediction.

We used our VarCap pipeline (https://github.com/ma2o/VarCap) (53) to detect single nucleotide and structural differences at high resolution and accuracy based on Illumina reads mapped to the reference *P. acanthamoebae* genome sequence. VarCap automatically performs quality filtering, mapping, variant calling, and postfiltering of the predicted variants. The output is a Variant Call Format (VCF) file with a detailed description of the variants as well as two PDF files, which give a graphical overview of variant coverage and their frequency distribution.

Zojer et al. (53) separately evaluated numerous variant detection tools for SNPs, insertions, and deletions, as well as structural variants. Owing to the different variant calling abilities of the separate tools at low frequencies, several tools were combined so as to increase the sensitivity of the VarCap pipeline. Furthermore, a variant call had to be supported by at least two different tools so as to gain precision and robustness. Combining all these selected software tools, all variants except inversions could be detected at a minimum read abundance of 2% at 400× total coverage, with a minimum of eight reads per variant, with high sensitivity.

### RNA-Seq Infection Experiments.

EBs were freshly purified from amoeba cultures grown in 500-cm^2^ culture flasks (Nalge Nunc International), in which EBs had been allowed to accumulate in the medium for 1 wk. These EBs originated from the end of the experiment of both the HT and VT regimes. Purification of EBs and subsequent quantification of purified EBs was carried out as described previously. Symbiont-free amoebae were harvested 3 d before infection and seeded at low cell density, followed by incubation at 20 °C until infection. To optimize infection efficiency, particularly at early time points, we used an MOI of 185 for 2 hpi, an MOI of 100 for 24 hpi, and an MOI of 25 for released EBs accumulated at 7 d postinfection. Precultivated amoebae were harvested, transferred to 50-mL Greiner tubes (Greiner Bio-One GmbH), and purified *Parachlamydia* EBs were added, followed by repeated centrifugation (centrifuged at 130 × *g* twice for 5 min each time and then once for 10 min at 20 °C) with vortexing between the centrifugation steps. Infected amoebae were then transferred back to the culture flasks and incubated in TSY medium at 20 °C for 2 h before the infection was synchronized by gently washing the attached amoebae three times with PAS. TSY medium was added to the cultures; some culture flasks were sampled at the 2 hpi time point, whereas the remaining culture flasks were incubated at 20 °C for 24 h. Released *Parachlamydia* EBs were harvested from the medium supernatant after 1 wk of incubation from amoeba–endosymbiont cultures that were grown at the same conditions and purified as described previously. All infection experiments were performed in biological triplicates.

### RNA Extraction and Sequencing.

In order to increase the coverage of the *Parachlamydia* transcriptome, a protocol for enrichment of bacteria prior to RNA extraction was employed (33). Each sample was processed in less than 7 min so as to minimize possible changes of the transcriptomes during enrichment, since the half-life of total mRNA from *E. coli* was demonstrated to be in this range (54). Infected amoebae were harvested and collected (7,600 × *g*, 2 min, 20 °C), after which the pellets were resuspended in a sucrose buffer (35 mM Tris-HCl, 250 mM sucrose, 25 mM KCl, 10 mM MgCl_2_) supplemented with 50 µg/mL rifampicin (Sigma-Aldrich Handels GmbH) in order to inhibit active transcription during the enrichment procedure (55, 56). Amoebae were then disrupted by vortexing in the presence of glass beads (diameter of 0.75 to 1 mm; Carl Roth) for 1 min. The suspensions were subsequently filtered through a 5-µm filter, the flowthrough fractions containing the bacteria were collected by centrifugation (10,600 × *g*, 2 min, room temperature), and the pellets were immediately resuspended in TRIzol reagent (Thermo Fisher Scientific). Extracellular *Parachlamydia* EBs were pelleted (20,800 × *g*, 2 min, room temperature); these pellets were then resuspended in sucrose buffer and treated like the enriched bacteria.

Cells were mechanically disrupted by bead beating for 30 s at 4.5 m/s using lysing matrix A tubes and a FastPrep-24 instrument (MP Biomedicals). Subsequent RNA extraction was performed according to the TRIzol guidelines. Residual DNA was then digested using the Turbo DNA-Free Kit (Thermo Fisher Scientific) as recommended by the manufacturer. DNase-treated RNA was precipitated with sodium acetate and ethanol and dissolved in nuclease-free water (Thermo Fisher Scientific), whereas DNA contamination was controlled for via PCR targeting a short region of the bacterial 16S rRNA gene (SigF2/R2 primers) (57) using 35 PCR cycles. rRNA was subsequently removed using the Ribo-Zero magnetic kit for gram-positive bacteria according to the manufacturer’s instructions (Illumina). For strand-specific cDNA library preparation, the NEBNext Ultra directional RNA library prep kit for Illumina, in combination with the NEBNext multiplex oligonucleotides (New England Biolabs), was used starting at first-strand cDNA synthesis. All libraries were sequenced using an Illumina HiSeq2500 system at the VBCF NGS unit with 50-bp read length.

### RNA-Seq Read Processing.

Sequencing reads were trimmed and cleaned before mapping (Dataset S10), based on sequencing read statistics obtained using Trimmomatic (v0.32) (50) and FastQC (v0.10.0) (58). Briefly, 1) the first 11 bases were removed; 2) any remaining adapter sequences were clipped; and 3) sequence reads shorter than 25 bases were removed, all done using Trimmomatic. To map bacterial reads to the *P. acanthamoebae* reference genome (27) BWA (v0.7.5a) (51) was used. Only unambiguously mapped reads were kept using SAMtools (v0.1.18) (52). Strand-specific reads per predicted gene were counted via HTSeq (v0.11.1) (59).

### Gene Expression Analyses.

Differentially expressed genes were determined between two consecutive time points (2 hpi to 24 hpi, 24 hpi to EBs, EBs to 2 hpi) using the R software environment (v3.6.1) and the Bioconductor package DESeq2 (v1.22.1) (60–62). Genes were considered differentially expressed if their expression changed twofold with a false-discovery rate (FDR) smaller than or equal to 0.05. Log_2_ fold changes (logFC) of differentially expressed genes and mean centered gene expression values (log_2_ transcripts per kilobase million [TPM]) were used for visualization as heat maps using the R package gplots (v3.0.1.1) (63).

To improve the available *P. acanthamoebae* genome annotation, for each gene we collected Pfam domains (bit score ≥25) (64), Kyoto Encyclopedia of Genes and Genomes (KEGG) pathway maps (65), Gene Ontology (GO) terms using Blast2GO (66), clusters of orthologous groups of proteins (COGs) and their functional categories using MaGe (67), and type III secretion effector predictions using Effective (68). The Bioconductor software package GOsEq. (69) was used to test for statistical enrichment of functional categories among different gene sets.

### Statistics.

Statistical analysis was performed using R (v3.6.1) (60). Details on sample sizes, in addition to the statistical tests conducted, are shown in the corresponding figure legends; the statistical parameters and significance are reported in the figure legends. Data were considered to be statistically significant when *P* ≤ 0.05. The fitness assays were performed on three to eight replicates, consisting of three technical replicates each for qPCR data. Comparisons for two groups were calculated by unpaired two-tailed Student’s *t* tests and the comparison for more than two groups was calculated by a Kruskal–Wallis test followed by Dunn’s multiple comparison post hoc test.

Three infection experiments (representing three time points) using different symbiont subpopulations were performed, and three samples for each time point and treatment (VT and HT) were used for RNA sequencing. We performed a number of statistical tests on the RNA-Seq data obtained in triplicate: a negative binomial model followed by a Wald test in DESeq2 (62) was used to identify differentially expressed genes between two consecutive time points for each treatment; a probability weighting function followed by a Wallenius approximation in GOsEq. (69) was used to test for statistical enrichment of functional categories (FDR ≤ 0.05) among different gene sets for each treatment; two-tailed Fisher’s exact tests were conducted to test whether predicted type III secreted proteins were significantly enriched in any given gene set for each treatment; and Kendall rank correlation was used to test the correlation between transcript abundance in the two treatments.

## Supplementary Material

Supplementary File

Supplementary File

Supplementary File

Supplementary File

Supplementary File

Supplementary File

Supplementary File

Supplementary File

Supplementary File

Supplementary File

Supplementary File

## Data Availability

All raw sequencing data were deposited in the National Center for Biotechnology Information (NCBI) database under bioproject accession no. PRJNA574613. RNA-Seq data can be accessed from the Gene Expression Omnibus (GEO) database through accession no. GSE138099.

## References

[1] Ewald P. W., Transmission modes and evolution of the parasitism-mutualism continuum. Ann. N. Y. Acad. Sci. 503, 295–306 (1987). 3304078 10.1111/j.1749-6632.1987.tb40616.x

[2] Michalakis Y., Olivieri I., Renaud F., Raymond M., Pleiotropic action of parasites: How to be good for the host. Trends Ecol. Evol. (Amst.) 7, 59–62 (1992). 21235952 10.1016/0169-5347(92)90108-N

[3] Hoeksema J. D.., A meta-analysis of context-dependency in plant response to inoculation with mycorrhizal fungi. Ecol. Lett. 13, 394–407 (2010). 20100237 10.1111/j.1461-0248.2009.01430.x

[4] Sachs J. L., Skophammer R. G., Regus J. U., Evolutionary transitions in bacterial symbiosis. Proc. Natl. Acad. Sci. U.S.A. 108 (suppl. 2), 10800–10807 (2011). 21690339 10.1073/pnas.1100304108 PMC3131820

[5] Lipsitch M., Siller S., Nowak M. A., The evolution of virulence in pathogens with vertical and horizontal transmission. Evolution 50, 1729–1741 (1996). 28565576 10.1111/j.1558-5646.1996.tb03560.x

[6] Yamamura N., Evolution of mutualistic symbiosis: A differential equation model. Res. Popul. Ecol. (Kyoto) 38, 211–218 (1996).

[7] Bright M., Bulgheresi S., A complex journey: Transmission of microbial symbionts. Nat. Rev. Microbiol. 8, 218–230 (2010). 20157340 10.1038/nrmicro2262 PMC2967712

[8] Frank S. A., Models of symbiosis. Am. Nat. 150 (suppl. 1), S80–S99 (1997). 18811314 10.1086/286051

[9] Yamamura N., Vertical transmission and evolution of mutualism from parasitism. Theor. Popul. Biol. 44, 95–109 (1993).

[10] Moran N. A., McCutcheon J. P., Nakabachi A., Genomics and evolution of heritable bacterial symbionts. Annu. Rev. Genet. 42, 165–190 (2008). 18983256 10.1146/annurev.genet.41.110306.130119

[11] Axelrod R., Hamilton W. D., The evolution of cooperation. Science 211, 1390–1396 (1981). 7466396 10.1126/science.7466396

[12] Anderson R. M., May R. M., Coevolution of hosts and parasites. Parasitology 85, 411–426 (1982). 6755367 10.1017/s0031182000055360

[13] Bull J. J., Rice W. R., Distinguishing mechanisms for the evolution of co-operation. J. Theor. Biol. 149, 63–74 (1991). 1881147 10.1016/s0022-5193(05)80072-4

[14] Turner P. E., Cooper V. S., Lenski R. E., Tradeoff between horizontal and vertical modes of transmission in bacterial plasmids. Evolution 52, 315–329 (1998). 28568337 10.1111/j.1558-5646.1998.tb01634.x

[15] Fine P. E. M., Vectors and vertical transmission: An epidemiologic perspective. Ann. N. Y. Acad. Sci. 266, 173–194 (1975). 829470 10.1111/j.1749-6632.1975.tb35099.x

[16] Ewald P. W., Host-Parasite relations, vectors, and the evolution of disease severity. Annu. Rev. Ecol. Syst. 14, 465–485 (1983).

[17] Bull J. J., Perspective: Virulence. Evolution 48, 1423–1437 (1994). 28568406 10.1111/j.1558-5646.1994.tb02185.x

[18] May R. M., Anderson R. M., Epidemiology and genetics in the coevolution of parasites and hosts. Proc. R. Soc. Lond. B Biol. Sci. 219, 281–313 (1983). 6139816 10.1098/rspb.1983.0075

[19] Bull J. J., Molineux I. J., Rice W. R., Selection of benevolence in a host-parasite system. Evolution 45, 875–882 (1991). 28564051 10.1111/j.1558-5646.1991.tb04356.x

[20] Herre E. A., Population structure and the evolution of virulence in nematode parasites of fig wasps. Science 259, 1442–1445 (1993). 17801279 10.1126/science.259.5100.1442

[21] Ewald P. W., Evolution of Infectious Disease, (Oxford University Press, 1994).

[22] Messenger S. L., Molineux I. J., Bull J. J., Virulence evolution in a virus obeys a trade-off. Proc. Biol. Sci. 266, 397–404 (1999). 10097397 10.1098/rspb.1999.0651 PMC1689683

[23] Stewart A. D., Logsdon J. M. Jr., Kelley S. E., An empirical study of the evolution of virulence under both horizontal and vertical transmission. Evolution 59, 730–739 (2005). 15926685

[24] Sachs J. L., Wilcox T. P., A shift to parasitism in the jellyfish symbiont *Symbiodinium microadriaticum*. Proc. Biol. Sci. 273, 425–429 (2006). 16615208 10.1098/rspb.2005.3346 PMC1560209

[25] Horn M.., Illuminating the evolutionary history of chlamydiae. Science 304, 728–730 (2004). 15073324 10.1126/science.1096330

[26] Abdelrahman Y. M., Belland R. J., The chlamydial developmental cycle. FEMS Microbiol. Rev. 29, 949–959 (2005). 16043254 10.1016/j.femsre.2005.03.002

[27] Collingro A.., Unity in variety—the pan-genome of the *Chlamydiae*. Mol. Biol. Evol. 28, 3253–3270 (2011). 21690563 10.1093/molbev/msr161 PMC3247790

[28] Horn M., *Chlamydiae* as symbionts in eukaryotes. Annu. Rev. Microbiol. 62, 113–131 (2008). 18473699 10.1146/annurev.micro.62.081307.162818

[29] Ebert D., The epidemiology and evolution of symbionts with mixed-mode transmission. Annu. Rev. Ecol. Evol. Syst. 44, 623–643 (2013).

[30] Magalon H., Nidelet T., Martin G., Kaltz O., Host growth conditions influence experimental evolution of life history and virulence of a parasite with vertical and horizontal transmission. Evolution 64, 2126–2138 (2010). 20163449 10.1111/j.1558-5646.2010.00974.x

[31] Dusi E., Gougat-Barbera C., Berendonk T. U., Kaltz O., Long-term selection experiment produces breakdown of horizontal transmissibility in parasite with mixed transmission mode. Evolution 69, 1069–1076 (2015). 25756600 10.1111/evo.12638

[32] Krzywinski M.., Circos: An information aesthetic for comparative genomics. Genome Res. 19, 1639–1645 (2009). 19541911 10.1101/gr.092759.109 PMC2752132

[33] König L.., Biphasic metabolism and host interaction of a chlamydial symbiont. mSystems 2, e00202-16 (2017). 28593198 10.1128/mSystems.00202-16 PMC5451489

[34] Omsland A., Sager J., Nair V., Sturdevant D. E., Hackstadt T., Developmental stage-specific metabolic and transcriptional activity of *Chlamydia trachomatis* in an axenic medium. Proc. Natl. Acad. Sci. U.S.A. 109, 19781–19785 (2012). 23129646 10.1073/pnas.1212831109 PMC3511728

[35] Mäurer A. P., Mehlitz A., Mollenkopf H. J., Meyer T. F., Gene expression profiles of *Chlamydophila pneumoniae* during the developmental cycle and iron depletion-mediated persistence. PLoS Pathog. 3, e83 (2007). 17590080 10.1371/journal.ppat.0030083 PMC1894823

[36] Betts-Hampikian H. J., Fields K. A., The chlamydial type III secretion mechanism: Revealing cracks in a tough nut. Front. Microbiol. 1, 114 (2010). 21738522 10.3389/fmicb.2010.00114 PMC3125583

[37] Subtil A., Collingro A., Horn M., Tracing the primordial chlamydiae: Extinct parasites of plants? Trends Plant Sci. 19, 36–43 (2014). 24210739 10.1016/j.tplants.2013.10.005

[38] Yao J.., Type II fatty acid synthesis is essential for the replication of *Chlamydia trachomatis*. J. Biol. Chem. 289, 22365–22376 (2014). 24958721 10.1074/jbc.M114.584185 PMC4139244

[39] Sixt B. S.., Metabolic features of *Protochlamydia amoebophila *elementary bodies—a link between activity and infectivity in *Chlamydiae*. PLoS Pathog. 9, e1003553 (2013). 23950718 10.1371/journal.ppat.1003553 PMC3738481

[40] Omsland A., Sixt B. S., Horn M., Hackstadt T., Chlamydial metabolism revisited: Interspecies metabolic variability and developmental stage-specific physiologic activities. FEMS Microbiol. Rev. 38, 779–801 (2014). 24484402 10.1111/1574-6976.12059 PMC4790414

[41] Elwell C., Mirrashidi K., Engel J., *Chlamydia *cell biology and pathogenesis. Nat. Rev. Microbiol. 14, 385–400 (2016). 27108705 10.1038/nrmicro.2016.30 PMC4886739

[42] Ebert D., Mangin K. L., The influence of host demography on the evolution of virulence of a microsporidian gut parasite. Evolution 51, 1828–1837 (1997). 28565099 10.1111/j.1558-5646.1997.tb05106.x

[43] Alizon S., Hurford A., Mideo N., Van Baalen M., Virulence evolution and the trade-off hypothesis: History, current state of affairs and the future. J. Evol. Biol. 22, 245–259 (2009). 19196383 10.1111/j.1420-9101.2008.01658.x

[44] Acevedo M. A., Dillemuth F. P., Flick A. J., Faldyn M. J., Elderd B. D., Virulence-driven trade-offs in disease transmission: A meta-analysis. Evolution 73, 636–647 (2019). 30734920 10.1111/evo.13692

[45] Jeon K. W., Ahn T. I., Temperature sensitivity: A cell character determined by obligate endosymbionts in amoebas. Science 202, 635–637 (1978). 17754039 10.1126/science.202.4368.635

[46] Minks A. K., Harrewijn P., Aphids: Their Biology, Natural Enemies, and Control, (Elsevier Science Limited, 1987).

[47] Lienard J.., Development of a new *Chlamydiales*-specific real-time PCR and its application to respiratory clinical samples. J. Clin. Microbiol. 49, 2637–2642 (2011). 21562107 10.1128/JCM.00114-11 PMC3147821

[48] Schmitz-Esser S.., The genome of the amoeba symbiont “*Candidatus* Amoebophilus asiaticus” reveals common mechanisms for host cell interaction among amoeba-associated bacteria. J. Bacteriol. 192, 1045–1057 (2010). 20023027 10.1128/JB.01379-09 PMC2812958

[49] Schmieder R., Edwards R., Quality control and preprocessing of metagenomic datasets. Bioinformatics 27, 863–864 (2011). 21278185 10.1093/bioinformatics/btr026 PMC3051327

[50] Bolger A. M., Lohse M., Usadel B., Trimmomatic: A flexible trimmer for Illumina sequence data. Bioinformatics 30, 2114–2120 (2014). 24695404 10.1093/bioinformatics/btu170 PMC4103590

[51] Li H., Durbin R., Fast and accurate short read alignment with Burrows-Wheeler transform. Bioinformatics 25, 1754–1760 (2009). 19451168 10.1093/bioinformatics/btp324 PMC2705234

[52] Li H..; 1000 Genome Project Data Processing Subgroup, The sequence alignment/map format and SAMtools. Bioinformatics 25, 2078–2079 (2009). 19505943 10.1093/bioinformatics/btp352 PMC2723002

[53] Zojer M.., Variant profiling of evolving prokaryotic populations. PeerJ 5, e2997 (2017). 28224054 10.7717/peerj.2997 PMC5316281

[54] Selinger D. W., Saxena R. M., Cheung K. J., Church G. M., Rosenow C., Global RNA half-life analysis in *Escherichia coli *reveals positional patterns of transcript degradation. Genome Res. 13, 216–223 (2003). 12566399 10.1101/gr.912603 PMC420366

[55] Dreses-Werringloer U., Padubrin I., Zeidler H., Köhler L., Effects of azithromycin and rifampin on *Chlamydia trachomatis* infection in vitro. Antimicrob. Agents Chemother. 45, 3001–3008 (2001). 11600348 10.1128/AAC.45.11.3001-3008.2001 PMC90774

[56] Sarov I., Becker Y., Deoxyribonucleic acid-dependent ribonucleic acid polymerase activity in purified trachoma elementary bodies: Effect of sodium chloride on ribonucleic acid transcription. J. Bacteriol. 107, 593–598 (1971). 5095283 10.1128/jb.107.3.593-598.1971 PMC246976

[57] Haider S., Collingro A., Walochnik J., Wagner M., Horn M., *Chlamydia*-like bacteria in respiratory samples of community-acquired pneumonia patients. FEMS Microbiol. Lett. 281, 198–202 (2008). 18312573 10.1111/j.1574-6968.2008.01099.x

[58] Andrews S., Fast QC: A quality control tool for high throughput sequence data. FastQC (2010). http://www.bioinformatics.babraham.ac.uk/projects/fastqc/. Accessed 22 November 2018.

[59] Anders S., Pyl P. T., Huber W., HTSeq—a Python framework to work with high-throughput sequencing data. Bioinformatics 31, 166–169 (2015). 25260700 10.1093/bioinformatics/btu638 PMC4287950

[60] R Development Core Team, R: A Language and Environment for Statistical Computing, (R Foundation for Statistical Computing, Vienna, Austria, 2011).

[61] Gentleman R. C.., Bioconductor: Open software development for computational biology and bioinformatics. Genome Biol. 5, R80 (2004). 15461798 10.1186/gb-2004-5-10-r80 PMC545600

[62] Love M. I., Huber W., Anders S., Moderated estimation of fold change and dispersion for RNA-seq data with DESeq2. Genome Biol. 15, 550 (2014). 25516281 10.1186/s13059-014-0550-8 PMC4302049

[63] Warnes G., gplots: various R programming tools for plotting data (R package Version 2, R Foundation for Statistical Computing, Vienna, Austria, 2005).

[64] El-Gebali S.., The Pfam protein families database in 2019. Nucleic Acids Res. 47, D427–D432 (2019). 30357350 10.1093/nar/gky995 PMC6324024

[65] Kanehisa M., Furumichi M., Tanabe M., Sato Y., Morishima K., KEGG: New perspectives on genomes, pathways, diseases and drugs. Nucleic Acids Res. 45, D353–D361 (2017). 27899662 10.1093/nar/gkw1092 PMC5210567

[66] Götz S.., High-throughput functional annotation and data mining with the Blast2GO suite. Nucleic Acids Res. 36, 3420–3435 (2008). 18445632 10.1093/nar/gkn176 PMC2425479

[67] Vallenet D.., MaGe: A microbial genome annotation system supported by synteny results. Nucleic Acids Res. 34, 53–65 (2006). 16407324 10.1093/nar/gkj406 PMC1326237

[68] Jehl M.-A., Arnold R., Rattei T., Effective—a database of predicted secreted bacterial proteins. Nucleic Acids Res. 39, D591–D595 (2011). 21071416 10.1093/nar/gkq1154 PMC3013723

[69] Young M. D., Wakefield M. J., Smyth G. K., Oshlack A., Gene ontology analysis for RNA-seq: Accounting for selection bias. Genome Biol. 11, R14 (2010). 20132535 10.1186/gb-2010-11-2-r14 PMC2872874

