# Supplementary material for: Molecular causes of an evolutionary shift along the parasitism–mutualism continuum in a bacterial symbiont
Source: Proc Natl Acad Sci U S A. 2020 Aug 19;117(35):21658–66. doi: 10.1073/pnas.2005536117 (PMC7474615; doi:10.1073/pnas.2005536117)
Supplement: Supplementary File [file pnas.2005536117.sapp.pdf]

Supporting Information

SI Text

Calculating the number of symbiont generations in each regime.

In order to put the observed evolutionary changes into context, it was important to calculate the total number of symbiont generations over the course of the experiment as best as possible. This would allow us to compare the number of generations between HT and VT regimes, since a large difference between the two values may improve our understanding of any observed changes.

In the VT treatment, TSY medium was replaced daily and this removed any released EBs present in the medium. Although these EBs were removed from the flask, thus preventing their ability to infect host cells, they still contributed to the total number of symbiont generations. We quantified the amount of EBs present in the medium per day over the course of three successive days with qPCR. We also quantified the amount of symbionts present in a fully infected continuous culture with freshly replaced medium with qPCR. In this way we were able to calculate the number of generations arising from released EBs per day. Apart from this, we measured the growth rate of amoeba infected with symbionts from this VT treatment. Since bacteria are transmitted vertically from parent to daughter cell, the doubling of amoeba cells would be equivalent to doubling of bacteria. We then added the number of generations arising from the released EBs to the number of generations arising from host cell division to calculate the total number of symbiont generations for the VT regime, which equated to 525 generations for the entire duration of the evolution experiment.

In the HT treatment, symbionts isolated from the medium were added weekly to naive amoebae. By quantifying the number of symbionts added at the start of the week and the number of symbionts in the medium at the end of the week, both with qPCR, we were able to calculate the number of symbiont generations arising from released EBs throughout the week. We also measured the growth rate of amoeba infected with

www.pnas.org/cgi/doi/10.1073/pnas.2005536117

symbionts from this HT treatment. By adding the two values together we were able to calculate the total number of symbiont generations for the HT regime, which equated to 560 generations for the entire duration of the evolution experiment.

Thus, we can say that the observed evolutionary changes in the HT and VT regimes have not simply been brought about as a result of a large difference between the total numbers of symbiont generations.

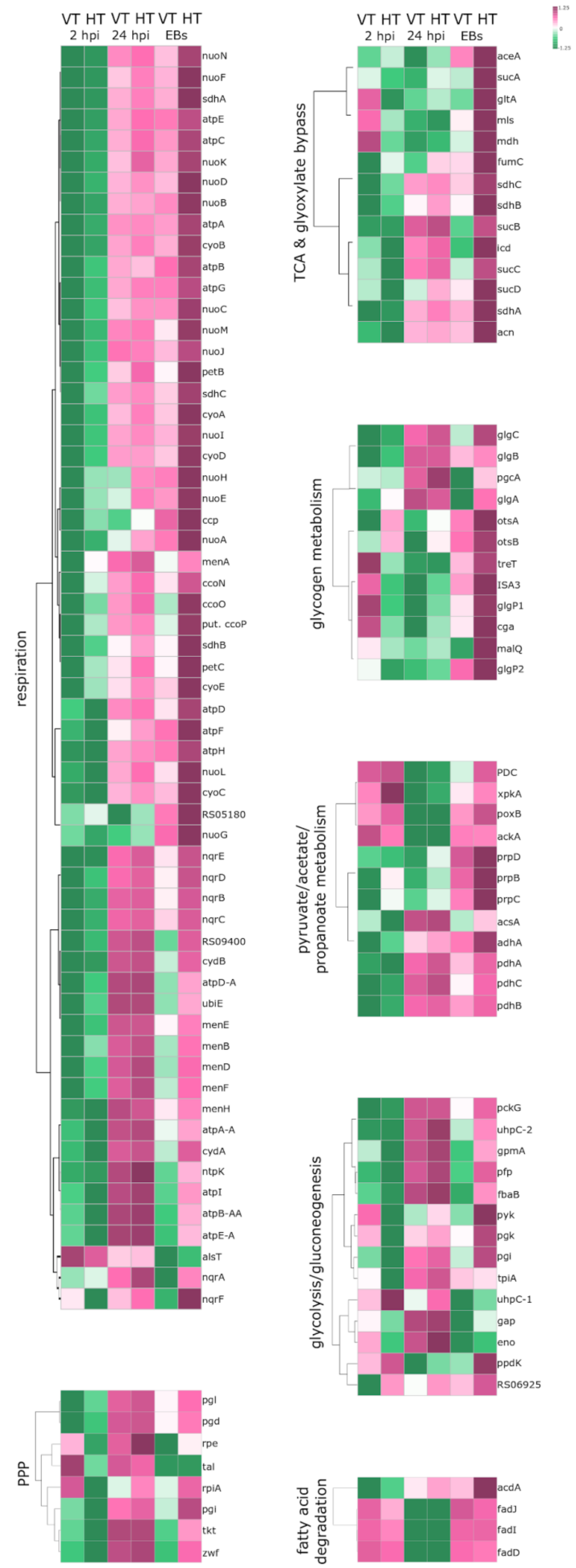
VT HT VT HT VT HT
2 hpi 24 hpi EBs
respiration
nuoN
nuoF
sdhA
atpE
atpC
nuoK
nuoD
nuoB
atpA
cyoB
atpB
atpG
nuoC
nuoM
nuoJ
petB
sdhC
cyoA
nuoI
cyoD
nuoH
nuoE
ccp
nuoA
menA
ccoN
ccoO
put. ccoP
sdhB
petC
cyoE
atpD
atpF
atpH
nuoL
cyoC
RS05180
nuoG
nqrE
nqrD
nqrB
nqrC
RS09400
cydB
atpD-A
ubiE
menE
menB
menD
menF
menH
atpA-A
cydA
ntpK
atpI
atpB-AA
atpE-A
alsT
nqrA
nqrF
PPP
pgl
pgd
rpe
tal
rpiA
pgi
tkt
zwf
TCA & glyoxylate bypass
aceA
sucA
gltA
mls
mdh
fumC
sdhC
sdhB
sucB
icd
sucC
sucD
sdhA
acn
glycogen metabolism
glgC
glgB
pgcA
glgA
otsA
otsB
treT
ISA3
glgP1
cga
malQ
glgP2
pyruvate/acetate/
propanoate metabolism
PDC
xpkA
poxB
ackA
prpD
prpB
prpC
acsA
adhA
pdhA
pdhC
pdhB
glycolysis/gluconeogenesis
pckG
uhpC-2
gpmA
pfp
fbaB
pyk
pgk
pgi
tpiA
uhpC-1
gap
eno
ppdK
RS06925
fatty acid
degradation
acdA
fadJ
fadI
fadD

Fig. S1 Heat maps of pathways involved in central carbon metabolism and energy generation.

Heat maps of RNA-Seq data from mean centered gene expression (TPM) of symbiont genes (listed in Dataset S4) in HT and VT regimes at all three time points during infection. All known loci involved or putatively involved in the pathways shown are depicted. Gene names or locus tags (without the prefix PUV_) are indicated. Highly expressed genes are shown in pink, whereas low gene expression is shown in green. The stronger the color, the stronger the gene expression value.

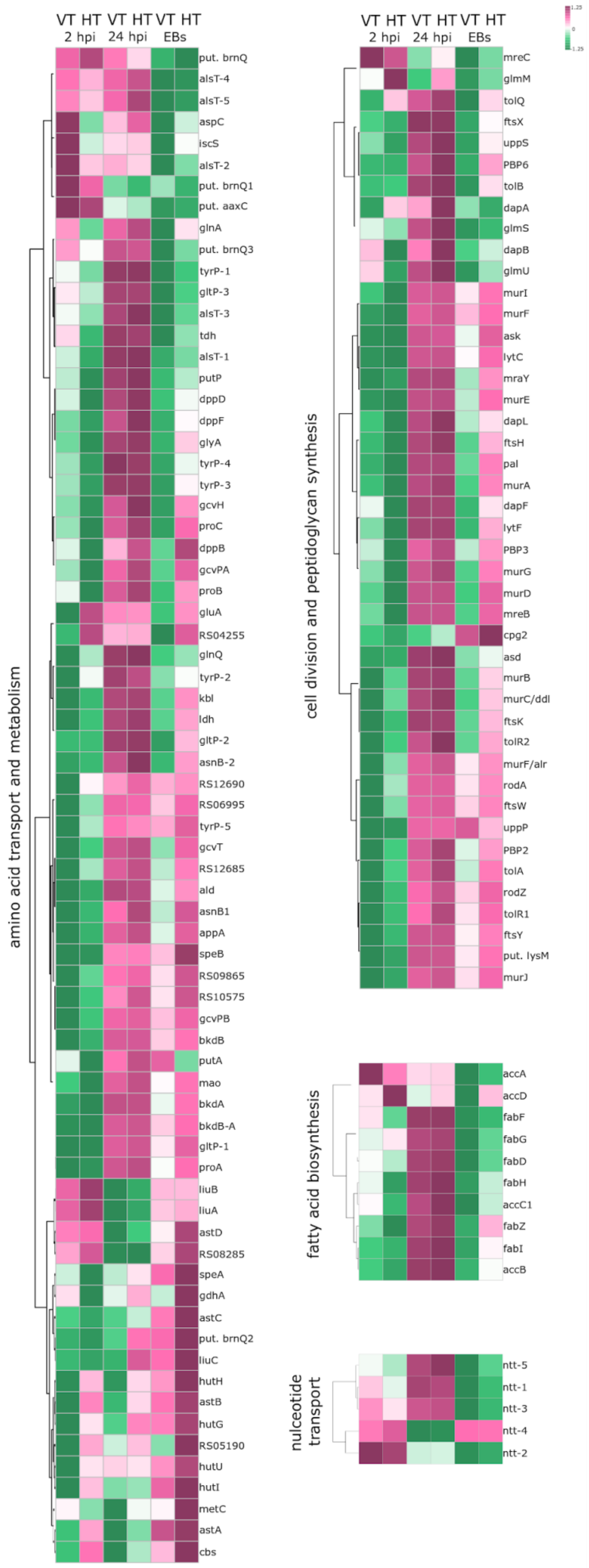

VT HT VT HT VT HT
2 hpi 24 hpi EBs
amino acid transport and metabolism
cell division and peptidoglycan synthesis
fatty acid biosynthesis
nulceotide transport
put. brnQ
alsT-4
alsT-5
aspC
iscS
alsT-2
put. brnQ1
put. aaxC
glnA
put. brnQ3
tyrP-1
gltP-3
alsT-3
tdh
alsT-1
putP
dppD
dppF
glyA
tyrP-4
tyrP-3
gcvH
proC
dppB
gcvPA
proB
gluA
RS04255
glnQ
tyrP-2
kbl
ldh
gltP-2
asnB-2
RS12690
RS06995
tyrP-5
gcvT
RS12685
ald
asnB1
appA
speB
RS09865
RS10575
gcvPB
bkdB
putA
mao
bkdA
bkdB-A
gltP-1
proA
liuB
liuA
astD
RS08285
speA
gdhA
astC
put. brnQ2
liuC
hutH
astB
hutG
RS05190
hutU
hutI
metC
astA
cbs
mreC
glmM
tolQ
ftsX
uppS
PBP6
tolB
dapA
glmS
dapB
glmU
murI
murF
ask
lytC
mraY
murE
dapL
ftsH
pal
murA
dapF
lytF
PBP3
murG
murD
mreB
cpg2
asd
murB
murC/ddl
ftsK
tolR2
murF/alr
rodA
ftsW
uppP
PBP2
tolA
rodZ
tolR1
ftsY
put. lysM
murJ
accA
accD
fabF
fabG
fabD
fabH
accC1
fabZ
fabI
accB
ntt-5
ntt-1
ntt-3
ntt-4
ntt-2

Fig. S2 Heat maps of metabolic pathways strongly upregulated at the peak of RB activity (i.e. at 24 hpi).

Heat maps of RNA-Seq data from mean centered gene expression (TPM) of symbiont genes (listed in Dataset S4) in HT and VT regimes at all three time points during infection. All known loci involved or putatively involved in the pathways shown are depicted. Gene names or locus tags (without the prefix PUV_) are indicated. Highly expressed genes are shown in pink, whereas low gene expression is shown in green. The stronger the color, the stronger the gene expression value.

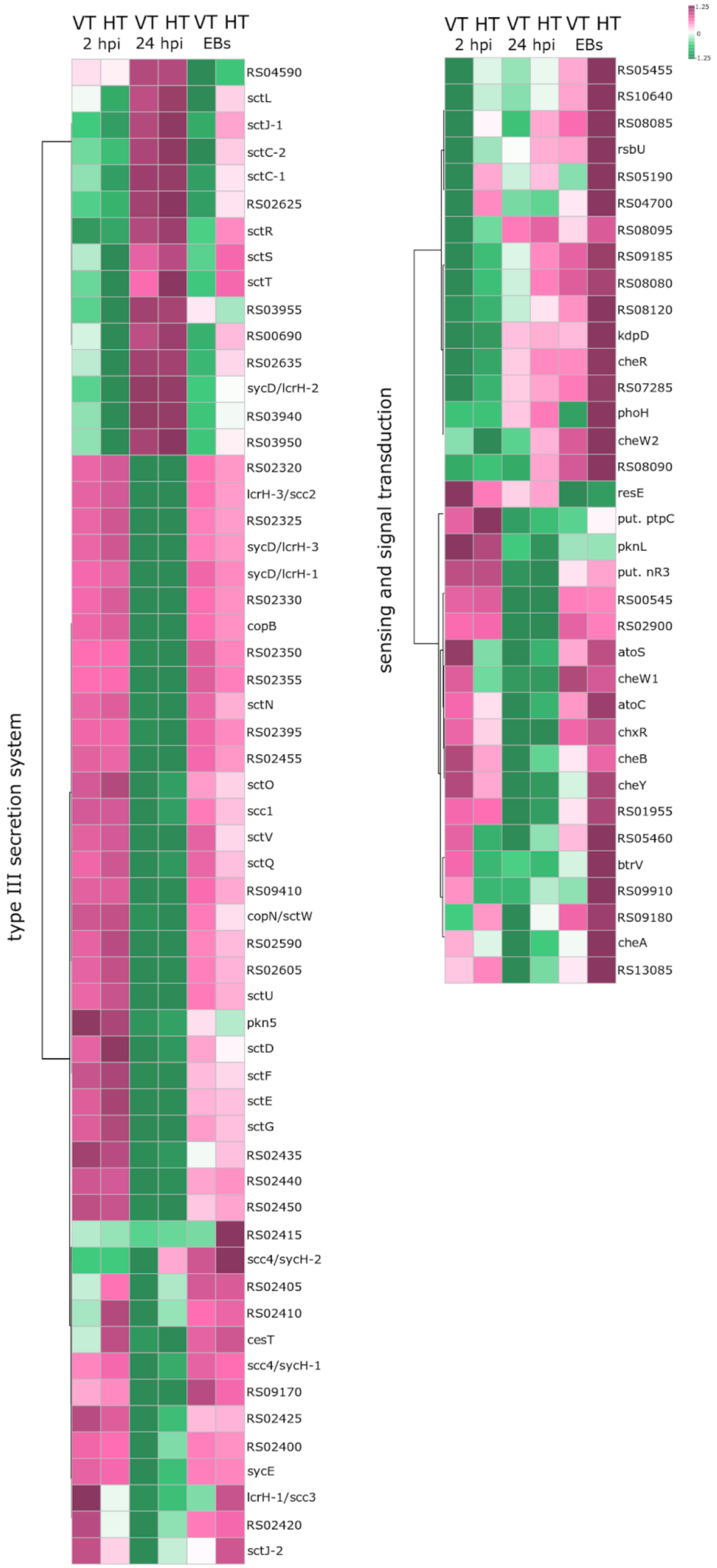
VT HT VT HT VT HT
2 hpi 24 hpi EBs
type III secretion system
RS04590
sctL
sctJ-1
sctC-2
sctC-1
RS02625
sctR
sctS
sctT
RS03955
RS00690
RS02635
sycD/lcrH-2
RS03940
RS03950
RS02320
lcrH-3/scc2
RS02325
sycD/lcrH-3
sycD/lcrH-1
RS02330
copB
RS02350
RS02355
sctN
RS02395
RS02455
sctO
scc1
sctV
sctQ
RS09410
copN/sctW
RS02590
RS02605
sctU
pkn5
sctD
sctF
sctE
sctG
RS02435
RS02440
RS02450
RS02415
scc4/sycH-2
RS02405
RS02410
cesT
scc4/sycH-1
RS09170
RS02425
RS02400
sycE
lcrH-1/scc3
RS02420
sctJ-2
VT HT VT HT VT HT
2 hpi 24 hpi EBs
sensing and signal transduction
RS05455
RS10640
RS08085
rsbU
RS05190
RS04700
RS08095
RS09185
RS08080
RS08120
kdpD
cheR
RS07285
phoH
cheW2
RS08090
resE
put. ptpC
pknL
put. nR3
RS00545
RS02900
atoS
cheW1
atoC
chxR
cheB
cheY
RS01955
RS05460
btrV
RS09910
RS09180
cheA
RS13085

Fig. S3 Heat maps of pathways involved in infection, sensing and signal transduction pathways.

Heat maps of RNA-Seq data from mean centered gene expression (TPM) of symbiont genes (listed in Dataset S4) in HT and VT regimes at all three time points during infection. All known loci involved or putatively involved in the pathways shown are depicted. Gene names or locus tags (without the prefix PUV_) are indicated. Highly expressed genes are shown in pink, whereas low gene expression is shown in green. The stronger the color, the stronger the gene expression value.

Datasets

Dataset S1.xlsx All values of host and symbiont fitness for individual replicates.

Dataset S2.xlsx Summary of all standing genetic variants and novel variants identified in the HT and VT populations.

Dataset S3.xlsx The percentage of the 112 unique genes differentially expressed in the HT symbiont population affected per functional category.

Dataset S4.xlsx Summary of normalized expression for genes from all replicates and time points of the HT and VT treatments. TPM=(reads mapping to gene)/(length of gene/1000)/(total reads per kilobase/1000000).

Dataset S5.xlsx Summary of all differentially expressed genes between two consecutive time points in both treatments. Genes were considered differentially expressed if their expression changed twofold with a false-discovery rate (FDR) smaller or equal 0.05.

Dataset S6.xlsx Summary of all 354 and 248 differentially expressed genes in HT and VT, respectively.

Dataset S7.xlsx Summary of all 112 and 8 uniquely differentially expressed genes in HT and VT, respectively.

Dataset S8.xlsx All individual $\log_2$ fold changes of genes that were differentially expressed between released EBs and the 2 hpi (false discovery rate of 0.05) for those

metabolic pathways and T3SS loci with marked differences between the HT and VT treatments.

Dataset S9.xlsx Summary of overrepresented functional categories (FDR $\leq$ 0.05) among differentially expressed genes between two consecutive time points for each set-up.

Dataset S10.xlsx RNA-Seq and read mapping statistics.
